# Health, Disability, and Economic Inactivity Following a Diagnosis of a Severe Mental Illness: Cohort Study of Electronic Health Records Linked at the Individual-Level, to Census from England

**DOI:** 10.1093/schbul/sbae195

**Published:** 2024-11-28

**Authors:** L Cybulski, M E Dewey, R Hildersley, C Morgan, R Stewart, M Wuerth, J Das-Munshi

**Affiliations:** Department of Psychological Medicine, King’s College London, Institute of Psychiatry Psychology and Neuroscience, London, SE5 8AF, United Kingdom; Division of Insurance Medicine, Karolinska Institutet, 171 77 Stockholm, Sweden; Health Service and Population Research Department, Institute of Psychiatry, Psychology and Neuroscience, King’s College London, London, SE5 8AF, United Kingdom; Department of Psychological Medicine, King’s College London, Institute of Psychiatry Psychology and Neuroscience, London, SE5 8AF, United Kingdom; Health Service and Population Research Department, Institute of Psychiatry, Psychology and Neuroscience, King’s College London, London, SE5 8AF, United Kingdom; Centre for Society & Mental Health, King’s College London, London, United Kingdom;; Department of Psychological Medicine, King’s College London, Institute of Psychiatry Psychology and Neuroscience, London, SE5 8AF, United Kingdom; South London and Maudsley NHS Foundation Trust, London, United Kingdom; Department of Psychological Medicine, King’s College London, Institute of Psychiatry Psychology and Neuroscience, London, SE5 8AF, United Kingdom; Department of Psychological Medicine, King’s College London, Institute of Psychiatry Psychology and Neuroscience, London, SE5 8AF, United Kingdom; Centre for Society & Mental Health, King’s College London, London, United Kingdom;; Population Heath Improvement UK (PHI-UK), United Kingdom;; South London and Maudsley NHS Foundation Trust, London, United Kingdom

**Keywords:** schizophrenia, bipolar affective disorders, disability, unemployment, self-rated health, census linkage

## Abstract

**Background:**

The association of social and clinical indicators with employment, disability, and health outcomes among individuals with severe mental illnesses (SMI) remains unclear. Existing evidence primarily comes from smaller cohort studies limited by shorter follow-up and high attrition, or registry-based research, which lacks information on important social determinants.

**Study Design:**

We utilized a novel data linkage consisting of clinical records of individuals diagnosed with schizophrenia-spectrum or bipolar disorders from the South London and Maudsley Mental Health Trust, linked at the individual-level to the 2011 UK Census, a rich source for sociodemographic information. Using logistic regression, we estimated adjusted odds ratios (aORs) and 95% confidence intervals to determine associations between socioeconomic and clinical indicators and economic inactivity, self-rated health, and disability outcomes.

**Results:**

The sample comprised 8249 people with SMI diagnoses. Economic inactivity (77.3%), disability (68.3%) and poor health (61.1%) were highly prevalent. Longer duration of illness and comorbid substance misuse were associated with economic inactivity, poorer self-rated health, and disability, with associations noted between living alone and all outcomes (aORs and 95% CI: Economic inactivity: 1.72, 1.45-2.03; disability: 1.48, 1.31-1.68; poor health: 1.32, 1.18-1.49). Relative to the White British group, Black African, South Asian, and Other Black groups were more likely to be economically inactive. Black Caribbean and other groups were less likely to report poorer self-rated health or disability.

**Conclusions:**

Our findings highlight considerable disability, poorer health, and economic inactivity experienced by people with SMI. Addressing comorbid substance misuse and social isolation could play a role in improving outcomes.

## Introduction

Schizophrenia and bipolar spectrum disorder are severe mental illnesses (SMIs) associated with significant morbidity and functional impairment. Compared with the general population, individuals with these disorders are more likely to be diagnosed with other disabling health conditions^1,2^ and experience reductions in life expectancy of up to 15 years.^3–5^ Similarly stark inequities have also been observed in terms of labor market participation. The likelihood of employment, itself an important indicator for social inclusion, functioning, and recovery, is generally low in this population, with estimates varying between 10% and 29% in the UK,^6,7^ far below the national average of 70% observed at equivalent time points.^8^ Existing studies have identified several factors that are likely to account for the substantial morbidity in this population, including smoking,^9^ substance misuse,^10^ obesity,^11^ and other health-related behaviors that increase the risk for somatic health problems.^1^ There is also evidence that individuals in more deprived communities experience poorer health outcomes,^3^ but comparatively little work has been done in characterizing the social determinants of health outcomes in this group, and factors associated with employment have rarely been the focus of larger investigations.

Traditional follow-up of recruited cohorts are typically limited by short periods of follow-up, relatively small sample sizes, and challenges of selective attrition. Cohorts derived from routine healthcare data circumvent many of these limitations but are constrained by the information that is available in clinical patient records. For example, individual-level data on housing tenure, living arrangement, socioeconomic status, education, migration, and other relevant sociodemographic measures are typically absent from a patient’s clinical record. Similarly, employment status is generally not recorded reliably. To address these issues, we utilized a unique data resource consisting of clinical mental health records from a diverse urban catchment in south London, UK, linked to national Census data at the individual-level with detailed socioeconomic information.^12^ We set out to examine within a cohort of individuals with SMIs the distribution of social and clinical characteristics and their associations with economic inactivity, self-rated health, and disability.

## Method

### Data Source

We delineated a cohort of individuals with an SMI diagnosis using a dataset consisting of de-identified clinical records from the South London and Maudsley National Health Service Foundation Trust (SLaM) linked to the 2011 Census of England and Wales at the individual-level. We have previously described the characteristics of this dataset and the methods employed in linking its constituent parts.^12^ In brief, SLaM provides all specialist mental healthcare to a geographic catchment of four southeast London boroughs (Croydon, Lambeth, Lewisham, Southwark; around 1.3 million residents), which has adopted fully electronic records across all services from 2006, and enabled research use of de-identified data from the full record via its Clinical Record Interactive Search (CRIS) platform,^13^ thus providing longitudinal data on patient’s care. This clinical data resource was then linked to data from the 2011 Census of England and Wales. This linkage provided additional individual-level data on socioeconomic factors, including long-term economic inactivity, disability, health status, tenure, ethnicity, migration, and other relevant socioeconomic measures. Of note, all data sourced from the Census applied to a single point in time (March 23rd, 2011), whereas clinical data from CRIS were collected during a patient’s contact with services up until the date of the Census.

### Study Participants

We identified individuals aged 16 or above who had been diagnosed with schizophrenia-spectrum or bipolar affective disorder before 23 March 2011. We chose this date as cutoff point because our outcome data was derived exclusively from the Census portion of the dataset, which was collected on this date. We included individuals with ICD-10 diagnoses of F20-F29 (schizophrenia, schizotypal, and delusional disorder) and F30 and F31(bipolar disorder). We excluded individuals if they had been diagnosed with a dementia or an organic brain disorder (F00-F09) prior to their schizophrenia or bipolar disorder diagnosis, or if they had died prior to Census. We also extracted data from a comparator non-SMI sample consisting of individuals who responded to the 2011 Census and who at the time had no recorded history of contact with SLaM and who lived in the four South London boroughs which constituted its catchment area. The inclusion of this comparator non-SMI sample allowed us to directly compare the prevalence of the study’s 3 outcomes and sociodemographic correlates.

### Measures

The study’s three primary outcomes were derived from the linked Census data. Outcomes for the present analysis were employment status, self-rated health, and disability. We ascertained employment status by cross-referencing Census variables that conveyed information about employment status. We considered an individual to be employed if they had indicated that they had worked 15 hours or more in the preceding week. Students, retirees, and individuals not in employment were considered to be economically inactive. Health status and disability were determined from 2 ordinal self-reported measures from Census. Presence of disability was ascertained with the question “Are your day-to-day activities limited because of a health problem or disability which has lasted, or expected to last, at least 12 months?” to which respondents could provide 3 answers: “no disability,” “limited a little,” and “limited a lot.” Health status was determined with the question “How is your health in general?” and respondents were asked to rate their health as “very good,” “good,” “fair,” “bad,” and “very bad.” We converted these 2 measures into binary outcomes where individuals who indicated that they were “limited a little” or “limited a lot” were considered to have a disability. Similarly, individuals who rated their health status to be worse than “fair” were considered to be in poor health.

For each outcome, we examined an array of sociodemographic and clinical indicators as exposures. All individual-level sociodemographic indicators were self-reported and derived from the Census and included sex, ethnicity, tenure, education, living arrangement (alone vs. not alone), marital status, and migration status. Information about age of onset, years since diagnosis, history of admission, substance misuse, and diagnosis type (i.e., affective vs. non-affective psychosis) came from cohort members’ clinical records. Within the SMI group, data on clinical characteristics were anchored to the date of clinical diagnosis. Information about the date of death was sourced from the ONS mortality registration, which was linked to our dataset. We determined years since diagnosis by subtracting the date of the Census from individual’s first date of mental health service contact. Diagnoses were ascribed by clinicians according to the 10th edition of the International Classification of Disease (ICD-10) with structured fields supplemented with information in the free text, derived using validated algorithms through natural language processing.^3^ We calculated age of onset by subtracting the date of birth from individuals’ first contact with mental health services and placed individuals in 3 categories (regular/early onset: 45 years or less; late onset 46-65; very late onset: 65 or more).^14^ Self-reported ethnicity was derived from Census and categorized according to the Office of National Statistics “18+1” classification^15^ into the following groups: “Black Caribbean,” “Black African,” “Black Other,” “White and Black Caribbean,” “Irish,” “White British” and “White other.” Bangladeshi, Indian, or Pakistani ethnicity was combined into a “South Asian” category. Individuals with a self-ascribed ethnicity that did not fit into these categories were placed in a miscellaneous “Other ethnicities” category.

In addition, a measure for area deprivation was derived through the Index of Multiple Deprivation (IMD) linked to postal address, taken from the clinical record. The IMD covers 7 domains of deprivation (income, employment, crime, barriers to housing, health and disability, living environment, and skills and training).^16^ IMD scores were provided at the Lower-layer Super Output Area (LSOA) level, which are geographical regions that correspond to approximately 400 to 1200 households. Scores were assigned according to individual’s postcode that was provided during their first contact with services and placed in quartile categories, with higher scores indicating higher levels of deprivation.

### Statistical Analyses

In order to provide a side-by-side comparison between those with and without an SMI diagnosis, we estimated the prevalence of outcomes and Census attributes in the comparator/non-SMI sample. We also examined how the prevalence of outcomes varied in both groups across all Census characteristics. This was done by fitting a generalized linear model with a binomial distribution and identity link to estimate risk differences, and then using the *margins* command in Stata to estimate marginal means to derive adjusted prevalence estimates for each of the variables. These prevalence estimates were adjusted for age and sex. We then fitted multivariable logistic regression models that were restricted to the SMI sample to estimate odds ratios (OR) to examine the association between sociodemographic (sex, age of onset, years since diagnosis, area level deprivation, tenure, education, ethnicity, living arrangement, marital status, and migration status) and clinical characteristics (history of mental health unit admission, comorbid alcohol and substance abuse, and diagnosis type (affective/non-affective)) and each outcome. We first examined crude associations (model A) and then estimates adjusted for sex, age of onset, years since diagnosis, education, tenure (i.e., rented vs. owned accommodation), and area level deprivation (Model B). When we estimated the association between diagnosis type (i.e., affective vs. non-affective) and employment, we chose not to adjust for education as it may be an intermediate step in the causal pathway between diagnosis type and employment, as prodromal symptoms may disrupt an individual’s education. To complement these analyses, we also fitted generalized linear models with a binomial distribution and identity link to derive risk differences for each outcome in order to provide a measure on the absolute scale, which can be accessed in the supplementary materials. Missing data was generally low, with the highest proportion of missingness observed for the qualifications variable (9.1%). All models were complete case analyses.

### Statistical Weights

Because the dataset we analyzed consisted of two datasets that had been linked (i.e., CRIS and Census), all models were weighted using inverse probability weights to mitigate against the potential bias introduced by records not matching in the construction of the linked dataset. In brief, we weighted observations inversely to their probability of being matched, so that observations, which were less likely to be matched received a higher weight. Since missingness, irrespective of matching status, was very low for the gender, age, and area level deprivation variables in CRIS, we adjusted for non-matching by applying inverse probability weights to all analyses, with these variables (gender, age, deprivation) specified as covariates for matching status (please see Cybulski et al., 2024 for more details).

## Results

**Figure 1.**
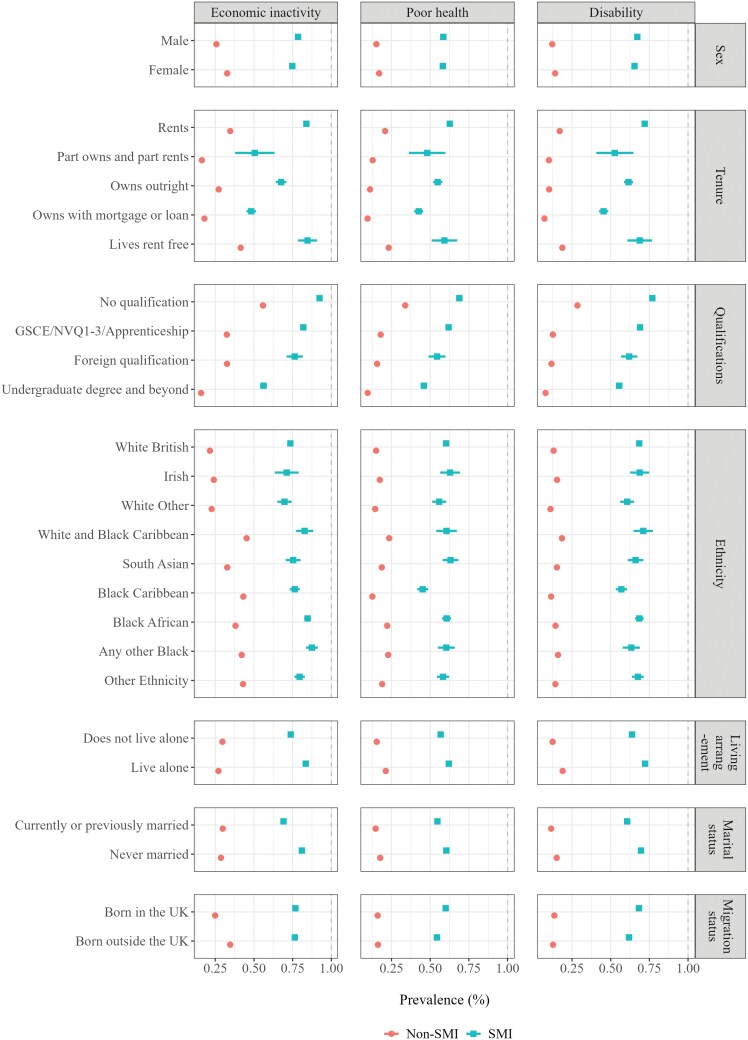
Age and Sex Adjusted Prevalence of Outcomes Among Individuals with and without a Severe Mental Illness (SMI) Diagnosis, by Census Attribute

After excluding individuals who did not meet study inclusion criteria (Supplementary Figure S1), 8249 were included in the SMI sample, of whom 2566 (31%) had an affective diagnosis. The average age of diagnosis was 41 years (standard deviation: 14.6) and there were slightly more men than women (51%). The majority (68%) lived in rented accommodation and close to a third had not obtained any educational qualifications (27%) and lived alone (31%). White British was the largest ethnic group (48%), followed by Black Caribbean (14%) and Black African (10%). Median time since diagnosis was 4.6 years (interquartile range: 2.3-7.5). Adverse outcomes were much more common among cohort members with an SMI, e.g., economic inactivity (76.9% vs. 29%), poor health (60.5% vs. 16.2%), and disability (67.4% vs. 13.3%) (Table 1). The sex and age distributions were similar in the two groups, whereas indicators of higher socioeconomic status (e.g., house ownership, higher degree of attained education, etc.), were more prevalent among individuals without an SMI. Sociodemographic characteristics that were more common among individuals with adverse outcomes in the SMI sample also tended to be more common among those in the non-SMI sample who had adverse outcomes. For example, the prevalence of poor self-rated health decreased incrementally with increasing level of education in both groups in a similar fashion, but the absolute age and sex adjusted prevalence estimates were substantially higher in the SMI group (please see Figure 1. for a detailed overview).

**Table 1.** Distribution of Outcomes and Census Attributes by Severe Mental Illness Diagnosis Status

	Diagnosis of a severe mental illness	
	Yes (*n* = 8249)	No (*n* = 596 124)
**Employment status**		
Employed	1515 (22.7)	332 495 (70.6)
Economically inactive	5153 (77.3)	138 520 (29.4)
**Disability status**		
No disability	2456 (31.7)	494 010 (86.7)
Disability	5295 (68.3)	75 649 (13.3)
**Health status**		
Good health	3099 (38.9)	486 893 (83.8)
Poor Health	4865 (61.1)	94 185 (16.2)
**Age band**		
15-24	581 (7.1)	98 999 (16.6)
25-34	1430 (17.4)	153 184 (25.7)
35-44	2034 (24.7)	119 991 (20.1)
45-54	1994 (24.2)	96 081 (16.1)
55-64	1221 (14.8)	60 609 (10.2)
65 or older	967 (11.8)	67 260 (11.3)
**Sex**		
Male	4160 (51.0)	288 095 (48.6)
Female	3994 (49.0)	304 777 (51.4)
**Tenure**		
Rents	5326 (68.2)	296 775 (51.7)
Part owns and part rents	73 (0.9)	7523 (1.3)
Owns outright	1129 (14.5)	88 876 (15.5)
Owns with mortgage or loan	1150 (14.7)	175 167 (30.5)
Lives rent free	128 (1.6)	5410 (0.9)
**Qualifications**		
No qualification	2080 (27.5)	81 605 (14.8)
GSCE/NVQ1-3/Apprenticeship	2675 (35.4)	147 592 (26.8)
Foreign qualification	358 (4.7)	50 832 (9.2)
Undergraduate degree and beyond	2437 (32.3)	271 168 (49.2)
**Ethnicity**		
White British	3936 (47.8)	263 739 (44.2)
Irish	209 (2.5)	13 629 (2.3)
White Other	505 (6.1)	74 862 (12.6)
White and Black Caribbean	233 (2.8)	8781 (1.5)
South Asian	350 (4.3)	34 251 (5.7)
Black Caribbean	842 (10.2)	67 868 (11.4)
Black African	1126 (13.7)	53 671 (9.0)
Any other Black	324 (3.9)	11 703 (2.0)
Other ethnicity	704 (8.6)	67 620 (11.3)
**Living arrangement**		
Does not live alone	5680 (69.0)	525 134 (88.1)
Live alone	2549 (31.0)	70 990 (11.9)
**Marital status**		
Currently or previously married	3514 (42.7)	313 897 (52.7)
Never married	4715 (57.3)	282 227 (47.3)
**Migration status**		
Born in the UK	5502 (66.9)	328 240 (55.1)
Born outside the UK	2727 (33.1)	267 884 (44.9)

### Economic Inactivity

As can be seen in Table 2, being diagnosed for 10 years or more (OR 2.14, 95%CI 1.28-3.57), never being married (1.66, 1.43-1.93), living alone (1.72, 1.45-2.03), and having a history of psychiatric hospital admission (1.79, 1.36-2.34), or substance misuse (1.93, 1.52-2.45) were strongly associated with economic inactivity after adjustment for sex, age of onset, years since diagnosis, education, tenure, and area level deprivation (Model B). Individual-level indicators of wealth or socioeconomic position were associated with reduced odds of economic inactivity. For example, all forms of property ownership were associated with better employment outcomes (e.g., Owning outright: 0.53, 0.43-0.66, Table 2: Model B), compared with renting. Similarly, individuals with any level of completed education were less likely to be economically inactive (e.g., GSCE/NVQ1-3/Apprenticeship: 0.39, 0.30-0.49, Table 2: Model B). Compared with the White British majority, individuals from South Asian, Black African, and Black Other ethnic groups were more likely to be economically inactive. Those diagnosed with an affective disorder had better employment outcomes (0.43, 0.38-0.50), relative to those with a non-affective diagnosis (Table 2: Model B). Results from our risk difference models were broadly consistent with our main analyses, although some characteristics (e.g., migration status) no longer distinguished individuals according to economic activity (Supplementary Table S2).

**Table 2.** Association Between Sociodemographic and Clinical Characteristics and Economic Inactivity

			Odds ratio (95% CI)	
	N (%)		Model A	Model B
Characteristics	Economically inactive	Employed	Unadjusted	Adjusted for sex, age of onset, years since diagnosis, area level deprivation, tenure, and level of qualification
**Sex**				
Male	2804 (54.9)	778 (50.7)	1.00 (ref)	1.00 (ref)
Female	2306 (45.1)	756 (49.3)	0.81 (0.72, 0.91)	1.02 (0.89, 1.17)
**Age of onset**				
Early (<45)	3809 (73.6)	1204 (78.3)	1.00 (ref)	1.00 (ref)
Mid (45-64)	1285 (24.8)	313 (20.4)	1.30 (1.13, 1.50)	1.39 (1.17, 1.64)
Late (65+)	78 (1.5)	21 (1.4)	1.43 (0.83, 2.46)	1.30 (0.62, 2.72)
**Years since diagnosis**				
Diagnosis in Census year	122 (2.4)	199 (12.9)	1.00 (ref)	1.00 (ref)
1-3	1511 (29.2)	574 (37.3)	0.90 (0.62, 1.29)	0.76 (0.48, 1.21)
4-6	1513 (29.3)	409 (26.6)	1.21 (0.84, 1.75)	0.89 (0.56, 1.41)
7-9	1242 (24.0)	277 (18.0)	1.56 (1.07, 2.28)	1.09 (0.68, 1.74)
10+	784 (15.2)	79 (5.1)	3.42 (2.24, 5.20)	2.14 (1.28, 3.57)
**IMD quartile**				
1 (Least deprived)	936 (18.8)	445 (29.8)	1.00 (ref)	1.00 (ref)
2	1207 (24.2)	421 (28.2)	1.37 (1.17, 1.60)	0.97 (0.80, 1.18)
3	1338 (26.8)	330 (22.1)	2.02 (1.71, 2.39)	1.26 (1.02, 1.54)
4 (Most deprived)	1504 (30.2)	299 (20.0)	2.46 (2.08, 2.92)	1.31 (1.07, 1.62)
**Tenure**				
Rents	3749 (77.0)	715 (47.3)	1.00 (ref)	1.00 (ref)
Part owns and part rents	32 (0.7)	30 (2.0)	0.19 (0.11, 0.31)	0.31 (0.18, 0.55)
Owns outright	499 (10.3)	228 (15.1)	0.41 (0.34, 0.49)	0.53 (0.43, 0.66)
Owns with mortgage or loan	496 (10.2)	526 (34.8)	0.17 (0.15, 0.20)	0.24 (0.20, 0.29)
Lives rent free	91 (1.9)	13 (0.9)	1.13 (0.64, 1.98)	1.35 (0.72, 2.53)
**Qualifications**				
No qualification	1428 (31.3)	95 (6.4)	1.00 (ref)	1.00 (ref)
GSCE/NVQ1-3/Apprenticeship	1826 (40.0)	434 (29.1)	0.31 (0.24, 0.39)	0.39 (0.30, 0.49)
Foreign qualification	183 (4.0)	58 (3.9)	0.22 (0.15, 0.31)	0.25 (0.17, 0.36)
Undergraduate degree +	1129 (24.7)	906 (60.7)	0.09 (0.07, 0.11)	0.12 (0.10, 0.16)
**Ethnicity**				
White British	2352 (45.5)	806 (53.2)	1.00 (ref)	1.00 (ref)
Irish	98 (1.9)	36 (2.4)	0.97 (0.65, 1.46)	0.85 (0.52, 1.37)
White Other	289 (5.6)	121 (8.0)	0.80 (0.63, 1.00)	0.89 (0.69, 1.17)
White and Black Caribbean	162 (3.1)	34 (2.2)	1.61 (1.09, 2.36)	0.98 (0.62, 1.53)
South Asian	224 (4.3)	70 (4.6)	1.12 (0.84, 1.49)	1.66 (1.20, 2.30)
Black Caribbean	533 (10.3)	166 (10.9)	1.12 (0.92, 1.36)	1.01 (0.79, 1.28)
Black African	777 (15.0)	133 (8.8)	2.10 (1.71, 2.58)	1.60 (1.25, 2.04)
Any other Black	256 (4.9)	37 (2.4)	2.52 (1.74, 3.64)	1.98 (1.25, 3.13)
Other ethnicity	481 (9.3)	113 (7.5)	1.44 (1.15, 1.81)	1.23 (0.94, 1.60)
**Living arrangement**				
Does not live alone	3452 (66.7)	1213 (80.0)	1.00 (ref)	1.00 (ref)
Lives alone	1720 (33.3)	303 (20.0)	2.00 (1.73, 2.31)	1.72 (1.45, 2.03)
**Marital status**				
Currently or previously married	1850 (35.8)	715 (47.2)	1.00 (ref)	1.00 (ref)
Never married	3322 (64.2)	801 (52.8)	1.60 (1.42, 1.81)	1.66 (1.43, 1.93)
**Migration status**				
Born in the UK	3577 (69.2)	1066 (70.3)	1.00 (ref)	1.00 (ref)
Born outside the UK	1595 (30.8)	450 (29.7)	1.03 (0.91, 1.18)	0.93 (0.80, 1.09)
**History of mental health unit admission**				
No	4726 (91.4)	1417 (93.5)	1.00 (ref)	1.00 (ref)
Yes	446 (8.6)	99 (6.5)	1.31 (1.04, 1.65)	1.79 (1.36, 2.34)
**Diagnosis type**				
Non-affective psychosis	3829 (74.0)	770 (50.8)	1.00 (ref)	1.00 (ref)
Affective psychosis	1343 (26.0)	746 (49.2)	0.36 (0.32, 0.40)	0.43 (0.38, 0.50)
**History of substance misuse**				
No	4340 (83.9)	1412 (93.1)	1.00 (ref)	1.00 (ref)
Yes	832 (16.1)	104 (6.9)	2.66 (2.14, 3.32)	1.93 (1.52, 2.45)

^a^We did not adjust for education when we estimated the effect of diagnosis type, as prodromal symptoms may interfere with educational achievement, it may therefore lie on the causal pathway between diagnosis type and employment.

### Disability and Health

Compared with economic inactivity, odds ratios tended to be smaller for disability and health outcomes, and there were some notable differences with respect to how the cohort’s characteristics were associated with these outcomes. For example, increasing area level deprivation was not associated with elevated odds for poor health or disability, and having a hospital admission did not distinguish between those who experienced either outcome after we adjusted for sex, age, and socioeconomic indicators (Tables 3 and 4; Model B). Otherwise the pattern was similar to those observed for education outcomes, with late onset (65 or older) (Disability: 1.92, 1.47-2.50); Poor health: 2.39, 1.87-3.04), being diagnosed for 10 years of more (Disability: 1.88, 1.30-2.71); Poor health: 1.54, 1.09-2.17), living alone (Disability: 1.48, 1.31-1.68; Poor health: 1.32, 1.18-1.49), and having a history of substance misuse (Disability: 1.32, 1.11-1.57; Poor health: 1.32, 1.13-1.55) emerging as the characteristics that were most strongly associated with poor health and disability (Tables 3 and 4; Model B). As with education, any form of house ownership was compared with renting associated with lower odds of disability and poor health, and the odds for each outcome decreased incrementally with rising levels of obtained education. Being diagnosed with an affective disorder was associated with lower odds of disability and poor health (Tables 3 and 4; Model B) and individuals born outside the UK were also less likely to report disability (0.71, 0.63-0.81) or poor health (0.81, 0.72-0.91) (Tables 3 and 4; Model B). In terms of ethnicity, the patterns differed compared with employment, as many minority ethnic groups were similarly or less likely to have a disability or to be of poor health compared with the White British reference group. For example, the Black Caribbean and Black African groups tended to have lower odds of poorer self-rated health and disability (Tables 3 and 4). As with employment, the patterning of results from the risk difference models was largely consistent with the results from the logistic regression models (Supplementary Tables S3 and S4).

**Table 3.** Association Between Sociodemographic and Clinical Characteristics and Poor Self-Rated Health

			Odds ratio (95% CI)	
	N (%)		Model A	Model B
Characteristics	Poor health	Good health	Unadjusted	Adjusted for sex, age of onset, years since diagnosis, area level deprivation, tenure, and level of qualification
**Sex**				
Male	2448 (50.6)	1617 (52.1)	1.00 (ref)	1.00 (ref)
Female	2391 (49.4)	1486 (47.9)	1.05 (0.96, 1.15)	1.08 (0.98, 1.20)
**Age of onset**				
Early (<45)	2931 (60.2)	2329 (74.7)	1.00 (ref)	1.00 (ref)
Mid (45-64)	1511 (31.0)	635 (20.4)	1.90 (1.70, 2.12)	1.86 (1.64, 2.10)
Late (65+)	426 (8.8)	152 (4.9)	2.25 (1.85, 2.74)	2.39 (1.87, 3.04)
**Years since diagnosis**				
Diagnosis in Census year	113 (2.3)	92 (3.0)	1.00 (ref)	1.00 (ref)
1-3	1462 (30.0)	1096 (35.2)	1.10 (0.82, 1.47)	1.03 (0.75, 1.42)
4-6	1465 (30.1)	944 (30.3)	1.30 (0.97, 1.75)	1.12 (0.81, 1.54)
7-9	1125 (23.1)	664 (21.3)	1.42 (1.05, 1.92)	1.26 (0.91, 1.75)
10+	703 (14.4)	320 (10.3)	1.83 (1.34, 2.50)	1.54 (1.09, 2.17)
**IMD quartile**				
1 (Least deprived)	975 (20.7)	720 (23.9)	1.00 (ref)	1.00 (ref)
2	1177 (25.0)	756 (25.1)	1.15 (1.01, 1.32)	0.99 (0.85, 1.15)
3	1231 (26.1)	748 (24.8)	1.21 (1.05, 1.38)	0.91 (0.78, 1.06)
4 (Most deprived)	1334 (28.3)	793 (26.3)	1.22 (1.07, 1.39)	0.89 (0.77, 1.04)
**Tenure**				
Rents	3408 (72.9)	1809 (60.7)	1.00 (ref)	1.00 (ref)
Part owns and part rents	37 (0.8)	35 (1.2)	0.53 (0.33, 0.85)	0.72 (0.43, 1.19)
Owns outright	677 (14.5)	437 (14.7)	0.84 (0.74, 0.97)	0.77 (0.66, 0.91)
Owns with mortgage or loan	480 (10.3)	647 (21.7)	0.40 (0.35, 0.45)	0.48 (0.41, 0.56)
Lives rent free	74 (1.6)	51 (1.7)	0.87 (0.59, 1.27)	0.82 (0.54, 1.22)
**Qualifications**				
No qualification	1527 (33.8)	520 (17.7)	1.00 (ref)	1.00 (ref)
GSCE/NVQ1-3/Apprenticeship	1617 (35.8)	1023 (34.9)	0.56 (0.49, 0.64)	0.67 (0.58, 0.78)
Foreign qualification	211 (4.7)	142 (4.8)	0.47 (0.37, 0.60)	0.52 (0.40, 0.66)
Undergraduate degree +	1160 (25.7)	1245 (42.5)	0.32 (0.28, 0.36)	0.39 (0.33, 0.44)
**Ethnicity**				
White British	2450 (50.3)	1407 (45.2)	1.00 (ref)	1.00 (ref)
Irish	139 (2.9)	61 (2.0)	1.31 (0.96, 1.80)	1.10 (0.77, 1.56)
White Other	286 (5.9)	204 (6.5)	0.78 (0.64, 0.95)	0.85 (0.69, 1.05)
White and Black Caribbean	129 (2.6)	92 (3.0)	0.81 (0.61, 1.08)	0.80 (0.58, 1.10)
South Asian	221 (4.5)	125 (4.0)	0.99 (0.78, 1.26)	1.18 (0.91, 1.53)
Black Caribbean	376 (7.7)	448 (14.4)	0.46 (0.39, 0.54)	0.47 (0.39, 0.57)
Black African	697 (14.3)	395 (12.7)	0.98 (0.85, 1.13)	0.84 (0.71, 0.98)
Any other Black	196 (4.0)	119 (3.8)	0.93 (0.73, 1.19)	0.90 (0.68, 1.19)
Other ethnicity	374 (7.7)	265 (8.5)	0.81 (0.68, 0.96)	0.86 (0.70, 1.04)
**Living arrangement**				
Does not live alone	3181 (65.3)	2296 (73.7)	1.00 (ref)	1.00 (ref)
Lives alone	1687 (34.7)	820 (26.3)	1.47 (1.33, 1.63)	1.32 (1.18, 1.49)
**Marital status**				
Currently or previously married	2117 (43.5)	1256 (40.3)	1.00 (ref)	1.00 (ref)
Never married	2751 (56.5)	1860 (59.7)	0.90 (0.82, 0.99)	1.09 (0.97, 1.22)
**Migration status**				
Born in the UK	3346 (68.7)	2088 (67.0)	1.00 (ref)	1.00 (ref)
Born outside the UK	1522 (31.3)	1028 (33.0)	0.89 (0.80, 0.98)	0.81 (0.72, 0.91)
**History of admission**				
No	4496 (92.4)	2834 (90.9)	1.00 (ref)	1.00 (ref)
Yes	372 (7.6)	282 (9.1)	0.83 (0.70, 0.99)	0.99 (0.82, 1.20)
**Diagnosis type**				
Non-affective psychosis	3399 (69.8)	2064 (66.2)	1.00 (ref)	1.00 (ref)
Affective psychosis	1469 (30.2)	1052 (33.8)	0.83 (0.76, 0.92)	1.00 (0.90, 1.12)
**History of substance misuse**				
No	4205 (86.4)	2778 (89.2)	1.00 (ref)	1.00 (ref)
Yes	663 (13.6)	338 (10.8)	1.34 (1.16, 1.55)	1.32 (1.13, 1.55)

**Table 4.** Association Between Sociodemographic and Clinical Characteristics and Self-Rated Disability

			Odds ratio (95% CI)	
	N (%)		Model A	Model B
Characteristics	Disability	No Disability	Unadjusted	Adjusted for sex, age of onset, years since diagnosis, area level deprivation, tenure, and level of qualification
**Sex**				
Male	2695 (51.2)	1251 (50.7)	1.00 (ref)	1.00 (ref)
Female	2573 (48.8)	1215 (49.3)	0.97 (0.87, 1.07)	1.06 (0.95, 1.18)
**Age of onset**				
Early (<45)	3313 (62.5)	1819 (73.6)	1.00 (ref)	1.00 (ref)
Mid (45-64)	1552 (29.3)	536 (21.7)	1.59 (1.42, 1.79)	1.56 (1.37, 1.78)
Late (65+)	434 (8.2)	117 (4.7)	2.01 (1.62, 2.49)	1.92 (1.47, 2.50)
**Years since diagnosis**				
Diagnosis in Census year	126 (2.4)	78 (3.2)	1.00 (ref)	1.00 (ref)
1-3	1553 (29.3)	962 (38.9)	1.05 (0.78, 1.42)	0.96 (0.69, 1.35)
4-6	1582 (29.9)	748 (30.3)	1.39 (1.03, 1.89)	1.15 (0.82, 1.61)
7-9	1266 (23.9)	465 (18.8)	1.81 (1.33, 2.46)	1.59 (1.13, 2.24)
10+	772 (14.6)	219 (8.9)	2.32 (1.67, 3.22)	1.88 (1.30, 2.71)
**IMD quartile**				
1 (Least deprived)	1081 (21.1)	589 (24.6)	1.00 (ref)	1.00 (ref)
2	1274 (24.8)	611 (25.5)	1.12 (0.98, 1.29)	0.90 (0.77, 1.06)
3	1329 (25.9)	590 (24.7)	1.21 (1.05, 1.39)	0.85 (0.73, 1.00)
4 (Most deprived)	1451 (28.3)	602 (25.2)	1.29 (1.12, 1.49)	0.83 (0.70, 0.97)
**Tenure**				
Rents	3756 (73.7)	1333 (56.1)	1.00 (ref)	1.00 (ref)
Part owns and part rents	37 (0.7)	33 (1.4)	0.41 (0.25, 0.66)	0.56 (0.34, 0.94)
Owns outright	715 (14.0)	367 (15.5)	0.70 (0.61, 0.81)	0.67 (0.57, 0.80)
Owns with mortgage or loan	507 (9.9)	604 (25.4)	0.29 (0.26, 0.34)	0.35 (0.30, 0.41)
Lives rent free	82 (1.6)	37 (1.6)	0.87 (0.57, 1.31)	0.87 (0.56, 1.35)
**Qualifications**				
No qualification	1641 (32.9)	369 (15.7)	1.00 (ref)	1.00 (ref)
GSCE/NVQ1-3/Apprenticeship	1782 (35.7)	827 (35.2)	0.50 (0.43, 0.57)	0.61 (0.52, 0.71)
Foreign qualification	221 (4.4)	122 (5.2)	0.39 (0.30, 0.51)	0.46 (0.35, 0.60)
Undergraduate degree +	1344 (26.9)	1031 (43.9)	0.29 (0.25, 0.34)	0.39 (0.34, 0.46)
**Ethnicity**				
White British	2674 (50.5)	1099 (44.5)	1.00 (ref)	1.00 (ref)
Irish	146 (2.8)	50 (2.0)	1.17 (0.83, 1.64)	1.02 (0.70, 1.48)
White Other	302 (5.7)	181 (7.3)	0.66 (0.54, 0.81)	0.73 (0.58, 0.91)
White and Black Caribbean	150 (2.8)	66 (2.7)	0.91 (0.67, 1.23)	0.81 (0.57, 1.14)
South Asian	224 (4.2)	109 (4.4)	0.82 (0.64, 1.05)	1.01 (0.78, 1.33)
Black Caribbean	452 (8.5)	345 (14.0)	0.52 (0.44, 0.61)	0.51 (0.42, 0.62)
Black African	737 (13.9)	313 (12.7)	0.97 (0.83, 1.13)	0.80 (0.67, 0.96)
Any other Black	199 (3.8)	104 (4.2)	0.74 (0.58, 0.96)	0.64 (0.48, 0.87)
Other ethnicity	415 (7.8)	205 (8.3)	0.84 (0.69, 1.01)	0.88 (0.71, 1.09)
**Living arrangement**				
Does not live alone	3413 (64.4)	1896 (76.7)	1.00 (ref)	1.00 (ref)
Lives alone	1886 (35.6)	576 (23.3)	1.80 (1.61, 2.01)	1.48 (1.31, 1.68)
**Marital status**				
Currently or previously married	2200 (41.5)	1081 (43.7)	1.00 (ref)	1.00 (ref)
Never married	3099 (58.5)	1391 (56.3)	1.11 (1.00, 1.22)	1.27 (1.13, 1.43)
**Migration status**				
Born in the UK	3668 (69.2)	1620 (65.5)	1.00 (ref)	1.00 (ref)
Born outside the UK	1631 (30.8)	852 (34.5)	0.81 (0.73, 0.90)	0.71 (0.63, 0.81)
**History of admission**				
No	4903 (92.5)	2227 (90.1)	1.00 (ref)	1.00 (ref)
Yes	396 (7.5)	245 (9.9)	0.72 (0.60, 0.85)	0.86 (0.70, 1.04)
**Diagnosis type**				
Non-affective psychosis	3695 (69.7)	1583 (64.0)	1.00 (ref)	1.00 (ref)
Affective psychosis	1604 (30.3)	889 (36.0)	0.76 (0.69, 0.85)	0.95 (0.84, 1.07)
**History of substance misuse**				
No	4576 (86.4)	2215 (89.6)	1.00 (ref)	1.00 (ref)
Yes	723 (13.6)	257 (10.4)	1.43 (1.22, 1.68)	1.32 (1.11, 1.57)

## Discussion

In this study, we utilized a novel dataset consisting of electronic mental health records from a diverse south London catchment linked to individual-level data from the 2011 UK Census to provide a unique perspective on the social determinants of outcomes in schizophrenia-spectrum and bipolar affective disorders. This is one of the first studies in England to use data from the Census at the individual-level linked to electronic mental health records and our findings highlight the considerable social exclusion and physical morbidity experienced by this population, as levels of economic inactivity, disability, and poor self-rated health were markedly elevated among individuals diagnosed with a severe mental illness.

Like previous studies,^6^ we observed a low employment rate among individuals diagnosed with a severe mental illness. Investigations from the UK,^6^ Norway,^17^ and Sweden^18^ that have focused on schizophrenia have reported proportions ranging from 3 to 10%. We observed a somewhat higher proportion in employment in our study, which may be explained by our estimates including individuals diagnosed with bipolar disorder, among whom employment rates typically are higher.^19^ Affective psychoses, such as bipolar disorder, are generally less disabling than non-affective psychoses. Negative symptoms (e.g., flat affect, social withdrawal, and loss of motivation), and the absence of effective treatments for them, have been highlighted as a potentially significant contributor for poor functioning in vocational or educational settings.^20^ However, longitudinal investigations of first episode psychosis indicate that only a minority of cases experience negative symptoms 10 years after the incident episode, whereas a majority continue to be unemployed.^21^ Thus, negative symptoms alone are therefore unlikely to be the main driver for labor market marginalization, and other factors such as stigma and discrimination may exert a greater influence over time. In terms of the correlates for economic inactivity that we examined, our findings are consistent with a recent register-based study from Sweden, in which comorbid substance misuse, prior hospitalization, older age, and being unmarried were associated with unemployment, whilst those with any degree of education were more likely to be employed.^18^ Some of these factors are likely to be indicators for the severity and course of the underlying illness. For example, individuals with completed education tend to exhibit shorter durations of diagnosis and higher levels of pre-morbid functioning, which are associated with better employment outcomes.^22,23^ Previous studies that have examined unemployment as an exposure for diagnosis have observed a higher prevalence among Black Caribbean and African individuals.^24,25^ In our study, Black Caribbean individuals did not differ from White British individuals, whilst those from Black African and Black Other backgrounds had worse employment outcomes. The latter 2 categories have rarely been examined in isolation in previous investigations, because of low numbers, and are typically included in one of the two other groups (e.g., Black Caribbean or Black Other), which may explain why our results are different, and highlight important differences in 'Black' groups which are frequently lost by aggregating groups together. The employment rate in the general population was in 2011 slightly lower among individuals from Black ethnic groups (e.g., ranging from 12% to 14%) relative to the other minority ethnic groups that we examined in this study^26^ and our findings may therefore reflect a continuation of pre-morbid inequities in labor market participation.

Our study showed that people living with SMI are more likely to report poorer health compared with non-SMI populations. Within the SMI population, poor self-rated health was associated with longer duration of illness, living alone, and with comorbid substance misuse. In general population studies, poorer self-rated health has been shown to be a powerful predictor of later mortality,^27^ independent of physical comorbidities, disability or underlying depressive mood states.^28^ Poorer self-rated health is also associated with fatal and non-fatal cardiovascular disease outcomes.^29^ Self-rated health may capture multiple dimensions of underlying health states, and although a subjective measure, has been noted as providing an accurate assessment of overall objective health status.^29^ In further work we plan to assess associations of this indicator in SMI populations with subsequent mortality, however the finding provides an insight into the possibility that people with SMI are more likely to make a negative appraisal of their underlying health states. This is consistent with what is known about the significant morbidity and reduced life expectancy in this population.^3–5^ The findings for self-rated disability in this sample, largely reflected this too.

Another factor that increasingly is being implicated in the pathogenesis of illnesses, including SMI, is social isolation. In our study, living alone was strongly linked with health and disability outcomes, sometimes even exceeding the effect sizes for comorbid substance misuse. Living alone for sustained periods, and other indicators for social isolation, such as the absence of friendships and not having regular contact with family members, have previously been linked with an increased risk for being diagnosed with a severe mental illness.^25^ Social isolation may also mediate the effect of other risk factors for poor health, such as substance misuse.^30^ Whilst we could not examine individual’s social networks, it is possible that these factors continue to adversely affect individuals in the period following diagnosis. Caution is nevertheless warranted when interpreting associations between living arrangements and outcomes, as individuals who live alone are not necessarily socially isolated and living alone may itself be a proxy measure for some other factor that may be causal, such as stigma and discrimination. In terms of ethnicity, the pattern for self-rated health and disability outcomes were broadly similar to each other. Our findings are broadly consistent with previous work that observed lower mortality in people from racially minoritised groups, which has been linked to residency in areas of higher own ethnic density,^31,32^ thought to confer relative benefits for well-being by buffering residents against social isolation.^33^

The use of Census-linked mental healthcare records allowed us to address some of the inherent methodological weaknesses in existing evidence from routine health records. Firstly, there is evidence that ethnicity recorded in healthcare settings is incomplete and inaccurate, with individuals from racially minoritised groups disproportionately affected.^34^ We circumvented this issue by using gold standard self-reported ethnicity data provided directly from respondents via Census. In most previous research based on electronic health records, the influence of social determinants such as education, tenure, employment, living arrangements, and migration status at the individual-level are never fully captured, and area level measures of deprivation have been used as a proxy, instead. This leads to a limited perspective on the influence of social determinants, despite a widespread acknowledgement that these indicators play a major role in the patterning of outcomes.^21^ By linking clinical records to the Census we had access to a rich array of individual-level social characteristics and other socioeconomic indicators, allowing us to directly assess how outcomes varied according to education, living situation, housing, and migration status. Our study used data from a large catchment area in London, UK, of approximately 1.3 million people, where the mental health service is the sole provider. This enhanced the representativeness of our findings, particularly with respect to racialized, and underserved communities, although findings may consequently generalize less to rural populations, or other countries.

Our study also had a number of limitations. For instance, because data from the Census was dated to 2011, our results reflect the sociodemographic distribution at that time. In addition, because all of our outcomes and some of our exposure information was sourced from the Census, they were all collected during a single point in time. This meant that we could not establish temporality between some exposures and outcomes. When creating the “South Asian” ethnic group category, we aggregated individuals who identified as Bangladeshi, Indian, and Pakistani, which may have obscured differences between these groups. However, the size of our sample also allowed us to examine other smaller ethnic groups (e.g., White and Black Caribbean and Black Other), which typically are aggregated into larger groups, potentially obscuring associations. In previous analyses,^12^ we demonstrated lower than expected levels of successful linkage of mental health records to Census, which could indicate that people with mental health conditions in contact with secondary mental healthcare may be less likely to participate in Census. Although this may be a concern, we addressed this by weighting estimates for non-matching to correct for this potential bias. Finally, a further limitation is that we did not include detail on psychotropic medications in the present analyses. We may plan to explore this in future research.

There has been scant large-scale research examining social determinants for outcomes among individuals diagnosed with SMIs. Through the use of a novel data linkage consisting of administrative and electronic mental health records, we were able to examine individual-level associations between sociodemographic and clinical factors and employment, self-rated health, and disability in people diagnosed with SMIs. Our findings demonstrate stark inequities with respect to these outcomes and suggests possible mechanisms (i.e., through social isolation, socioeconomic adversity, and lower education) that warrant further examination and which could be targeted to improve outcomes for this group of people.

## Supplementary material

Supplementary material is available at https://academic.oup.com/schizophreniabulletin/.

sbae195_suppl_Supplementary_Figure_S1_Tables_S2-S4

sbae195_suppl_Supplementary_Material
