# Supplementary material for: Health, Disability, and Economic Inactivity Following a Diagnosis of a Severe Mental Illness: Cohort Study of Electronic Health Records Linked at the Individual-Level, to Census from England
Source: Schizophr Bull. 2024 Nov 28;51(5):1367–79. doi: 10.1093/schbul/sbae195 (PMC12414549; doi:10.1093/schbul/sbae195)
Supplement: sbae195_suppl_Supplementary_Figure_S1_Tables_S2-S4 [file sbae195_suppl_supplementary_figure_s1_tables_s2-s4.docx]

**Figure S1**. Flow of study participants

52,376 observations in CRIS linked to CENSUS

596,124 individuals without a history of contact with mental health services appended to dataset

Exclusions:

- 35,668 did not have a severe mental illness diagnosis
- 8,211 diagnosed with a severe mental illness after the study end date on 23 March 2011
- 248 died prior to census

8,249 individuals included with a severe mental illness diagnosis

| **Table S1**. List of variables by dataset | | |  |
| --- | --- | --- | --- |
| **Variable** | **Description** | **Source** | **Date** |
| empstat | Employment status | 2011 Census | 23 March 2011 |
| hours | Number of hours worked last week | 2011 Census | 23 March 2011 |
| lastyrwork | Last year of work | 2011 Census | 23 March 2011 |
| disability | Self-rated disability status | 2011 Census | 23 March 2011 |
| health | Self-rated health status | 2011 Census | 23 March 2011 |
| age_cen | Age at Census | 2011 Census | 23 March 2011 |
| sex_cen | Sex | 2011 Census | 23 March 2011 |
| tenure | Tenure | 2011 Census | 23 March 2011 |
| quals | Highest obtained qualification | 2011 Census | 23 March 2011 |
| ethpuk | Self-ascribed ethnicity | 2011 Census | 23 March 2011 |
| live_alone | Living arrangement | 2011 Census | 23 March 2011 |
| marstat | Marital status | 2011 Census | 23 March 2011 |
| cob | Country of birth | 2011 Census | 23 March 2011 |
| age_of_onset | Age at first contact with mental health services | CRIS (health records) | N/A |
| years_since_diagnosis | Time elapsed since first contact up until Census date | CRIS (health records) | N/A |
| imd_quartile | Index of multiple deprivation quartile | CRIS (health records) | Based on postcode that was provided during first contact with services |
| hospital_admission | History of admission to psychiatric hospital | CRIS (health records) | Date of diagnosis |
| diagnosis_type | Affective vs. non-affective diagnosis | CRIS (health records) | Date of diagnosis |
| substance_misuse | History of comorbid substance misuse disorder | CRIS (health records) | Date of diagnosis |

| **Table S2.** Risk differences in economic activity outcomes among individuals with a SMI diagnosis | | | | |
| --- | --- | --- | --- | --- |
|  | **Economically active** | |  | |
|  | **No (N, %)** | **Yes (N, %)** | **Risk Difference (95% CI)** | |
| **Characteristics** |  |  |  |  |
| **Sex** |  |  | **Model A: Crude model** | **Model B: Sex, onset and years since diagnosis adjusted** |
| Male | 2804 (54.9) | 759 (50.2) | 0.00 (ref) | 0.00 (ref) |
| Female | 2306 (45.1) | 754 (49.8) | -0.04 (-0.06, -0.02) | -0.02 (-0.04, -0.00) |
| **Age of onset** |  |  |  |  |
| Early (< 45) | 3809 (73.6) | 1187 (78.3) | 0.00 (ref) | 0.00 (ref) |
| Mid (45-64) | 1285 (24.8) | 312 (20.6) | 0.04 (0.02, 0.07) | 0.05 (0.03, 0.07) |
| Late (65+) | 78 (1.5) | 17 (1.1) | 0.06 (-0.02, 0.14) | 0.04 (-0.03, 0.11) |
| **Years since diagnosis** |  |  |  |  |
| Diagnosis in Census year | 122 (2.4) | 44 (2.9) | 0.00 (ref) | 0.00 (ref) |
| 1-3 | 1511 (29.2) | 624 (41.2) | -0.02 (-0.09, 0.05) | -0.02 (-0.09, 0.05) |
| 4-6 | 1513 (29.3) | 459 (30.3) | 0.04 (-0.04, 0.11) | 0.04 (-0.04, 0.11) |
| 7-9 | 1242 (24.0) | 302 (19.9) | 0.08 (0.01, 0.15) | 0.08 (0.01, 0.16) |
| 10+ | 784 (15.2) | 87 (5.7) | 0.17 (0.10, 0.24) | 0.17 (0.10, 0.25) |
| **IMD quartile** |  |  |  |  |
| 1 (Least deprived) | 936 (18.8) | 443 (30.1) | 0.00 (ref) | 0.00 (ref) |
| 2 | 1207 (24.2) | 424 (28.8) | 0.06 (0.03, 0.10) | 0.05 (0.03, 0.08) |
| 3 | 1338 (26.8) | 316 (21.5) | 0.13 (0.10, 0.16) | 0.11 (0.10, 0.13) |
| 4 (Most deprived) | 1504 (30.2) | 289 (19.6) | 0.16 (0.13, 0.19) | 0.14 (.,.) * |
| **Tenure** |  |  |  |  |
| Rents | 3749 (77.0) | 693 (46.5) | 0.00 (ref) | 0.00 (ref) |
| Part owns and part rents | 32 (0.7) | 30 (2.0) | -0.34 (-0.47, -0.21) | -0.33 (-0.46, -0.20) |
| Owns outright | 499 (10.3) | 234 (15.7) | -0.16 (-0.19, -0.12) | -0.15 (-0.19, -0.11) |
| Owns with mortgage or loan | 496 (10.2) | 518 (34.8) | -0.36 (-0.40, -0.33) | -0.35 (-0.38, -0.31) |
| Lives rent free | 91 (1.9) | 15 (1.0) | 0.02 (-0.05, 0.08) | 0.00 (-0.06, 0.07) |
| **Qualifications** |  |  |  |  |
| No qualification | 1489 (31.7) | 97 (6.6) | 0.00 (ref) | 0.00 (ref) |
| GSCE/NVQ1-3/Apprenticeship | 1861 (39.6) | 420 (28.5) | -0.12 (-0.14, -0.10) | -0.11 (-0.13, -0.10) |
| Foreign qualification | 211 (4.5) | 63 (4.3) | -0.17 (-0.23, -0.12) | -0.16 (-0.21, -0.11) |
| Undergraduate degree + | 1135 (24.2) | 894 (60.7) | -0.37 (-0.40, -0.35) | -0.36 (-0.38, -0.33) |
| **Ethnicity** |  |  |  |  |
| White British | 2352 (45.5) | 806 (53.2) | 0.00 (ref) | 0.00 (ref) |
| Irish | 98 (1.9) | 36 (2.4) | -0.01 (-0.08, 0.07) | -0.01 (-0.09, 0.06) |
| White Other | 289 (5.6) | 121 (8.0) | -0.05 (-0.09, 0.00) | -0.02 (-0.07, 0.02) |
| White and Black Caribbean | 162 (3.1) | 34 (2.2) | 0.08 (0.02, 0.14) | 0.07 (0.02, 0.12) |
| South Asian | 224 (4.3) | 70 (4.6) | 0.02 (-0.03, 0.07) | 0.02 (-0.03, 0.07) |
| Black Caribbean | 533 (10.3) | 166 (10.9) | 0.02 (-0.02, 0.06) | 0.02 (-0.01, 0.05) |
| Black African | 777 (15.0) | 133 (8.8) | 0.11 (0.09, 0.14) | 0.10 ( ., .) |
| Any other Black | 256 (4.9) | 37 (2.4) | 0.14 (0.09, 0.18) | 0.11 (0.07, 0.14) |
| Other ethnicity | 481 (9.3) | 113 (7.5) | 0.06 (0.03, 0.10) | 0.05 (0.03, 0.08) |
| **Living arrangement** |  |  |  |  |
| Does not live alone | 3452 (66.7) | 1213 (80.0) | 0.00 (ref) | 0.00 (ref) |
| Lives alone | 1720 (33.3) | 303 (20.0) | 0.11 (0.09, 0.13) | 0.09 (.,.) * |
| **Marital status** |  |  |  |  |
| Currently or previously married | 1850 (35.8) | 715 (47.2) | 0.00 (ref) | 0.00 (ref) |
| Never married | 3322 (64.2) | 801 (52.8) | 0.08 (0.06, 0.11) | 0.10 (.,.) * |
| **Migration status** |  |  |  |  |
| Born in the UK | 3577 (69.2) | 1066 (70.3) | 0.00 (ref) | 0.00 (ref) |
| Born outside the UK | 1595 (30.8) | 450 (29.7) | 0.01 (-0.02, 0.03) | 0.00 (-0.02, 0.03) |
| **History of mental health unit admission** |  |  |  |  |
| No | 4726 (91.4) | 1417 (93.5) | 0.00 (ref) | 0.00 (ref) |
| Yes | 446 (8.6) | 99 (6.5) | 0.04 (0.01, 0.08) | 0.08 (0.05, 0.12) |
| **Diagnosis type** |  |  |  |  |
| Non-affective psychosis | 3829 (74.0) | 770 (50.8) | 0.00 (ref) | 0.00 (ref) |
| Affective psychosis | 1343 (26.0) | 746 (49.2) | -0.19 (-0.22, -0.17) | -0.17 (-0.20, -0.15) |
| **History of substance misuse** |  |  |  |  |
| No | 4340 (83.9) | 1412 (93.1) | 0.00 (ref) | 0.00 (ref) |
| Yes | 832 (16.1) | 104 (6.9) | 0.14 (0.11, 0.16) | 0.13 (.,.) * |
| * *(.,.) denotes failure in model convergence* | | | | |

| **Table S3.** Risk differences in self-rated disability outcomes among individuals with a SMI diagnosis | | | | |
| --- | --- | --- | --- | --- |
|  | **Disability** | |  | |
|  | **Yes (N, %)** | **No (N, %)** | **Risk Difference (95% CI)** | |
| **Characteristics** |  |  |  |  |
| **Sex** |  |  | **Model A: Crude model** | **Model B: Sex, onset and years since diagnosis adjusted** |
| Male | 2448 (50.6) | 1617 (52.1) | 0.00 (ref) | 0.00 (ref) |
| Female | 2391 (49.4) | 1486 (47.9) | 0.01 (-0.01, 0.03) | 0.01 (-0.02, 0.03) |
| **Age of onset** |  |  |  |  |
| Early (< 45) | 2931 (60.2) | 2329 (74.7) | 0.00 (ref) | 0.00 (ref) |
| Mid (45-64) | 1511 (31.0) | 635 (20.4) | 0.15 (0.12, 0.17) | 0.15 (0.12, 0.17) |
| Late (65+) | 426 (8.8) | 152 (4.9) | 0.18 (0.14, 0.22) | 0.20 (0.16, 0.24) |
| **Years since diagnosis** |  |  |  |  |
| Diagnosis in Census year | 113 (2.3) | 92 (3.0) | 0.00 (ref) | 0.00 (ref) |
| 1-3 | 1462 (30.0) | 1096 (35.2) | 0.02 (-0.05, 0.10) | 0.03 (-0.04, 0.10) |
| 4-6 | 1465 (30.1) | 944 (30.3) | 0.06 (-0.01, 0.14) | 0.06 (-0.01, 0.13) |
| 7-9 | 1125 (23.1) | 664 (21.3) | 0.09 (0.01, 0.16) | 0.10 (0.02, 0.17) |
| 10+ | 703 (14.4) | 320 (10.3) | 0.14 (0.07, 0.22) | 0.15 (0.08, 0.23) |
| **IMD quartile** |  |  |  |  |
| 1 (Least deprived) | 975 (20.7) | 720 (23.9) | 0.00 (ref) | 0.00 (ref) |
| 2 | 1177 (25.0) | 756 (25.1) | 0.03 (0.00, 0.07) | 0.04 (0.01, 0.07) |
| 3 | 1231 (26.1) | 748 (24.8) | 0.05 (0.01, 0.08) | 0.04 (0.01, 0.07) |
| 4 (Most deprived) | 1334 (28.3) | 793 (26.3) | 0.05 (0.02, 0.08) | 0.04 (0.01, 0.08) |
| **Tenure** |  |  |  |  |
| Rents | 3408 (72.9) | 1809 (60.7) | 0.00 (ref) | 0.00 (ref) |
| Part owns and part rents | 37 (0.8) | 35 (1.2) | -0.16 (-0.28, -0.04) | -0.14 (-0.26, -0.02) |
| Owns outright | 677 (14.5) | 437 (14.7) | -0.04 (-0.07, -0.01) | -0.07 (-0.10, -0.04) |
| Owns with mortgage or loan | 480 (10.3) | 647 (21.7) | -0.23 (-0.26, -0.19) | -0.21 (-0.24, -0.18) |
| Lives rent free | 74 (1.6) | 51 (1.7) | -0.03 (-0.12, 0.06) | -0.03 (-0.12, 0.06) |
| **Qualifications** |  |  |  |  |
| No qualification | 1527 (33.8) | 520 (17.7) | 0.00 (ref) | 0.00 (ref) |
| GSCE/NVQ1-3/Apprenticeship | 1617 (35.8) | 1023 (34.9) | -0.12 (-0.15, -0.10) | -0.09 (-0.11, -0.06) |
| Foreign qualification | 211 (4.7) | 142 (4.8) | -0.17 (-0.23, -0.11) | -0.15 (-0.20, -0.09) |
| Undergraduate degree + | 1160 (25.7) | 1245 (42.5) | -0.27 (-0.29, -0.24) | -0.23 (-0.26, -0.21) |
| **Ethnicity** |  |  |  |  |
| White British | 2450 (50.3) | 1407 (45.2) | 0.00 (ref) | 0.00 (ref) |
| Irish | 139 (2.9) | 61 (2.0) | 0.06 (-0.01, 0.13) | 0.02 (-0.04, 0.09) |
| White Other | 286 (5.9) | 204 (6.5) | -0.06 (-0.11, -0.01) | -0.04 (-0.09, 0.01) |
| White and Black Caribbean | 129 (2.6) | 92 (3.0) | -0.05 (-0.12, 0.02) | -0.02 (-0.09, 0.05) |
| South Asian | 221 (4.5) | 125 (4.0) | -0.00 (-0.06, 0.05) | 0.02 (-0.03, 0.07) |
| Black Caribbean | 376 (7.7) | 448 (14.4) | -0.19 (-0.23, -0.15) | -0.16 (-0.20, -0.13) |
| Black African | 697 (14.3) | 395 (12.7) | -0.00 (-0.04, 0.03) | -0.01 (-0.04, 0.03) |
| Any other Black | 196 (4.0) | 119 (3.8) | -0.02 (-0.07, 0.04) | -0.00 (-0.06, 0.05) |
| Other ethnicity | 374 (7.7) | 265 (8.5) | -0.05 (-0.09, -0.01) | -0.03 (-0.08, 0.01) |
| **Living arrangement** |  |  |  |  |
| Does not live alone | 3181 (65.3) | 2296 (73.7) | 0.00 (ref) | 0.00 (ref) |
| Lives alone | 1687 (34.7) | 820 (26.3) | 0.09 (0.07, 0.11) | 0.07 (0.04, 0.09) |
| **Marital status** |  |  |  |  |
| Currently or previously married | 2117 (43.5) | 1256 (40.3) | 0.00 (ref) | 0.00 (ref) |
| Never married | 2751 (56.5) | 1860 (59.7) | -0.02 (-0.05, -0.00) | 0.03 (0.01, 0.05) |
| **Migration status** |  |  |  |  |
| Born in the UK | 3346 (68.7) | 2088 (67.0) | 0.00 (ref) | 0.00 (ref) |
| Born outside the UK | 1522 (31.3) | 1028 (33.0) | -0.03 (-0.05, -0.01) | -0.05 (-0.08, -0.03) |
| **History of mental health unit admission** |  |  |  |  |
| No | 4496 (92.4) | 2834 (90.9) | 0.00 (ref) | 0.00 (ref) |
| Yes | 372 (7.6) | 282 (9.1) | -0.04 (-0.09, -0.00) | -0.00 (-0.04, 0.04) |
| **Diagnosis type** |  |  |  |  |
| Non-affective psychosis | 3399 (69.8) | 2064 (66.2) | 0.00 (ref) | 0.00 (ref) |
| Affective psychosis | 1469 (30.2) | 1052 (33.8) | -0.04 (-0.07, -0.02) | -0.04 (-0.06, -0.02) |
| **History of substance misuse** |  |  |  |  |
| No | 4205 (86.4) | 2778 (89.2) | 0.00 (ref) | 0.00 (ref) |
| Yes | 663 (13.6) | 338 (10.8) | 0.07 (0.04, 0.10) | 0.08 (0.05, 0.12) |
|  | | | | |

| **Table S4.** Risk differences in self-rated health outcomes among individuals with a SMI diagnosis | | | | |
| --- | --- | --- | --- | --- |
|  | **Poor self-rated health** | |  | |
|  | **Yes (N, %)** | **No (N, %)** | **Risk Difference (95% CI)** | |
| **Characteristics** |  |  |  |  |
| **Sex** |  |  | **Model A: Crude model** | **Model B: Sex, onset, and years since diagnosis adjusted** |
| Male | 2448 (50.6) | 1617 (52.1) | 0.00 (ref) | 0.00 (ref) |
| Female | 2391 (49.4) | 1486 (47.9) | 0.01 (-0.01, 0.03) | 0.01 (-0.02, 0.03) |
| **Age of onset** |  |  |  |  |
| Early (< 45) | 2931 (60.2) | 2329 (74.7) | 0.00 (ref) | 0.00 (ref) |
| Mid (45-64) | 1511 (31.0) | 635 (20.4) | 0.15 (0.12, 0.17) | 0.15 (0.12, 0.17) |
| Late (65+) | 426 (8.8) | 152 (4.9) | 0.18 (0.14, 0.22) | 0.20 (0.16, 0.24) |
| **Years since diagnosis** |  |  |  |  |
| Diagnosis in Census year | 113 (2.3) | 92 (3.0) | 0.00 (ref) | 0.00 (ref) |
| 1-3 | 1462 (30.0) | 1096 (35.2) | 0.02 (-0.05, 0.10) | 0.03 (-0.04, 0.10) |
| 4-6 | 1465 (30.1) | 944 (30.3) | 0.06 (-0.01, 0.14) | 0.06 (-0.01, 0.13) |
| 7-9 | 1125 (23.1) | 664 (21.3) | 0.09 (0.01, 0.16) | 0.10 (0.02, 0.17) |
| 10+ | 703 (14.4) | 320 (10.3) | 0.14 (0.07, 0.22) | 0.15 (0.08, 0.23) |
| **IMD quartile** |  |  |  |  |
| 1 (Least deprived) | 975 (20.7) | 720 (23.9) | 0.00 (ref) | 0.00 (ref) |
| 2 | 1177 (25.0) | 756 (25.1) | 0.03 (0.00, 0.07) | 0.04 (0.01, 0.07) |
| 3 | 1231 (26.1) | 748 (24.8) | 0.05 (0.01, 0.08) | 0.04 (0.01, 0.07) |
| 4 (Most deprived) | 1334 (28.3) | 793 (26.3) | 0.05 (0.02, 0.08) | 0.04 (0.01, 0.08) |
| **Tenure** |  |  |  |  |
| Rents | 3408 (72.9) | 1809 (60.7) | 0.00 (ref) | 0.00 (ref) |
| Part owns and part rents | 37 (0.8) | 35 (1.2) | -0.16 (-0.28, -0.04) | -0.14 (-0.26, -0.02) |
| Owns outright | 677 (14.5) | 437 (14.7) | -0.04 (-0.07, -0.01) | -0.07 (-0.10, -0.04) |
| Owns with mortgage or loan | 480 (10.3) | 647 (21.7) | -0.23 (-0.26, -0.19) | -0.21 (-0.24, -0.18) |
| Lives rent free | 74 (1.6) | 51 (1.7) | -0.03 (-0.12, 0.06) | -0.03 (-0.12, 0.06) |
| **Qualifications** |  |  |  |  |
| No qualification | 1527 (33.8) | 520 (17.7) | 0.00 (ref) | 0.00 (ref) |
| GSCE/NVQ1-3/Apprenticeship | 1617 (35.8) | 1023 (34.9) | -0.12 (-0.15, -0.10) | -0.09 (-0.11, -0.06) |
| Foreign qualification | 211 (4.7) | 142 (4.8) | -0.17 (-0.23, -0.11) | -0.15 (-0.20, -0.09) |
| Undergraduate degree + | 1160 (25.7) | 1245 (42.5) | -0.27 (-0.29, -0.24) | -0.23 (-0.26, -0.21) |
| **Ethnicity** |  |  |  |  |
| White British | 2450 (50.3) | 1407 (45.2) | 0.00 (ref) | 0.00 (ref) |
| Irish | 139 (2.9) | 61 (2.0) | 0.06 (-0.01, 0.13) | 0.02 (-0.04, 0.09) |
| White Other | 286 (5.9) | 204 (6.5) | -0.06 (-0.11, -0.01) | -0.04 (-0.09, 0.01) |
| White and Black Caribbean | 129 (2.6) | 92 (3.0) | -0.05 (-0.12, 0.02) | -0.02 (-0.09, 0.05) |
| South Asian | 221 (4.5) | 125 (4.0) | -0.00 (-0.06, 0.05) | 0.02 (-0.03, 0.07) |
| Black Caribbean | 376 (7.7) | 448 (14.4) | -0.19 (-0.23, -0.15) | -0.16 (-0.20, -0.13) |
| Black African | 697 (14.3) | 395 (12.7) | -0.00 (-0.04, 0.03) | -0.01 (-0.04, 0.03) |
| Any other Black | 196 (4.0) | 119 (3.8) | -0.02 (-0.07, 0.04) | -0.00 (-0.06, 0.05) |
| Other ethnicity | 374 (7.7) | 265 (8.5) | -0.05 (-0.09, -0.01) | -0.03 (-0.08, 0.01) |
| **Living arrangement** |  |  |  |  |
| Does not live alone | 3181 (65.3) | 2296 (73.7) | 0.00 (ref) | 0.00 (ref) |
| Lives alone | 1687 (34.7) | 820 (26.3) | 0.09 (0.07, 0.11) | 0.07 (0.04, 0.09) |
| **Marital status** |  |  |  |  |
| Currently or previously married | 2117 (43.5) | 1256 (40.3) | 0.00 (ref) | 0.00 (ref) |
| Never married | 2751 (56.5) | 1860 (59.7) | -0.02 (-0.05, -0.00) | 0.03 (0.01, 0.05) |
| **Migration status** |  |  |  |  |
| Born in the UK | 3346 (68.7) | 2088 (67.0) | 0.00 (ref) | 0.00 (ref) |
| Born outside the UK | 1522 (31.3) | 1028 (33.0) | -0.03 (-0.05, -0.01) | -0.05 (-0.08, -0.03) |
| **History of mental health unit admission** |  |  |  |  |
| No | 4496 (92.4) | 2834 (90.9) | 0.00 (ref) | 0.00 (ref) |
| Yes | 372 (7.6) | 282 (9.1) | -0.04 (-0.09, -0.00) | -0.00 (-0.04, 0.04) |
| **Diagnosis type** |  |  |  |  |
| Non-affective psychosis | 3399 (69.8) | 2064 (66.2) | 0.00 (ref) | 0.00 (ref) |
| Affective psychosis | 1469 (30.2) | 1052 (33.8) | -0.04 (-0.07, -0.02) | -0.04 (-0.06, -0.02) |
| **History of substance misuse** |  |  |  |  |
| No | 4205 (86.4) | 2778 (89.2) | 0.00 (ref) | 0.00 (ref) |
| Yes | 663 (13.6) | 338 (10.8) | 0.07 (0.04, 0.10) | 0.08 (0.05, 0.12) |
|  | | | | |
