# Supplementary figures and images for: Health, Disability, and Economic Inactivity Following a Diagnosis of a Severe Mental Illness: Cohort Study of Electronic Health Records Linked at the Individual-Level, to Census from England
Source: Schizophr Bull. 2024 Nov 28;51(5):1367–79. doi: 10.1093/schbul/sbae195 (PMC12414549; doi:10.1093/schbul/sbae195)

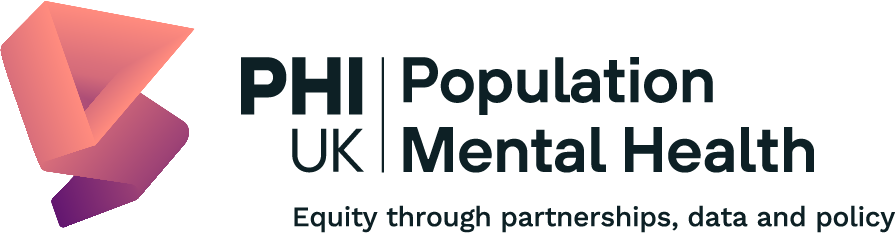

Supplement: sbae195_suppl_Supplementary_Material [file sbae195_suppl_supplementary_material.zip › image001.png]

Supplement: sbae195_suppl_Supplementary_Material [file sbae195_suppl_supplementary_material.zip › image003.gif]

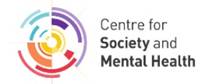

Supplement: sbae195_suppl_Supplementary_Material [file sbae195_suppl_supplementary_material.zip › image002.jpg]
